# Miniature semi-rigid ureteroscopy with holmium-yttrium-aluminium-garnet laser vs shockwave lithotripsy in the management of upper urinary tract stones >1 cm in children

**DOI:** 10.1080/2090598X.2020.1738105

**Published:** 2020-03-23

**Authors:** Mohamed Omran, Ahmed Sakr, Esam A. E. Desoky, Maged M. Ali, Mohamed M. H. Abdalla

**Affiliations:** Department of Urology, Faculty of Medicine, Zagazig University, Zagazig, Egypt

**Keywords:** Paediatric, ureteroscopy, shockwaves, laser, upper urinary tract

## Abstract

**Objective:**

To compare the efficacy and safety of miniature semi-rigid ureteroscopy (URS) with holmium (Ho)-yttrium-aluminium-garnet (YAG) laser lithotripsy vs shockwave lithotripsy (SWL) for treating upper urinary tract (UUT) calculi >1 cm in children.

**Patients and methods:**

Children with unilateral single UUT ureteric stones of >1 cm were prospectively enrolled in this study. Patients were randomly divided into two groups: Group 1, treated with SWL; and Group 2, treated with URS (6/7.5 F) and laser lithotripsy. The patients’ characteristics, stones demographics, operative time, adjunctive procedures, stone-free rate (SFR), re-treatment rate, and complications were statistically analysed and compared. Success was defined as stone-free status (no stone residual of ≥0.3 cm) at 1 month from the initial treatment without any auxiliary procedures.

**Results:**

In all, 68 patients with UUT stones met our inclusion criteria. There were no significant differences between the two groups for patient or stone demographics. In Group 1, the SFR was 26/34 (76.4%) and in Group 2 it was 33/34 (97.1%) (*P* = 0.03). A total of 12 auxiliary procedures in Group 1 and two in Group 2 were needed to reach a 100% SFR (*P* = 0.014). There were no significant differences between the two groups for operative times, adjunctive procedures, number of complicated cases or complications of Grade ≥III *(P* = 0.65, *P* = 0.23, *P* = 0.77, and *P* = 0.62, respectively).

**Conclusion:**

Miniature semi-rigid URS with Ho-YAG laser lithotripsy for UUT ureteric stones of >1 cm in children was more effective than SWL in terms of SFR and re-treatment rate, with no significant difference in the rate or grade of complications.

**Abbreviations:**

EQ: efficiency quotient; KUB: plain abdominal radiograph of the kidneys, ureters and bladder; RCT: randomised controlled trial; SFR: stone-free rate; SWL: shockwave lithotripsy; URS: ureteroscopy; US: ultrasonography/ultrasound; URS: ureteroscopy; UUT: upper urinary tract; YAG: yttrium-aluminium-garnet

## Introduction

Urinary stone disease in children is not an uncommon problem [1], which is likely recur due to many causes including metabolic disorders and congenital anatomical abnormality in the urinary tract [2,3].

This issue of recurrence dictates the adoption of minimally invasive treatment options in children with stone disease. For many years, shockwave lithotripsy (SWL) was effective and safe for treating children with upper urinary tract (UUT) calculi. It was embraced as the first-line treatment for renal stones of ≤2 cm and UUT ureteric stones of ≤1 cm [4]. As children appeared to be capable of clearing even larger stones without pre-SWL stenting, SWL can be used for stones >1 cm in the upper ureter [1]. Ureteroscopy (URS) for UUT urolithiasis has a higher success rate and a lower re-treatment rate when compared to SWL [5].

The development of flexible and smaller calibre semi-rigid ureteroscopes and introduction of lasers, as a lithotripsy energy source, have increased the ability of paediatric urologists to perform URS even in young children [3,6–8].

Therefore, in the present study, we compared the effectiveness [stone-free rate (SFR), adjunctive procedures, and re-treatment rates] and safety (complication rates) of miniature semi-rigid URS with holmium (Ho)-yttrium-aluminium-garnet (YAG) laser lithotripsy vs SWL in the management of UUT ureteric stones of >1 cm in children.

## Patients and methods

This prospective randomised comparative study was carried out at the Department of Urology, Zagazig University Hospital. Children (aged <16 years) with unilateral single radiopaque UUT ureteric stones of >1 cm, with densities of 500–1000 HU, were enrolled in this study. Patients with bilateral or multiple stones, congenital renal or ureteric anomalies and who failed SWL were excluded from the study. The study was approved by our Scientific and Ethics Committee (Institutional Review Board number 5789) and informed consent was obtained from all the patients’ parents. For all patients, urine analysis, urine culture and sensitivity, plain abdominal radiograph of the kidneys, ureters and bladder (KUB; to insure radio-opacity of the stone in all cases), renal ultrasonography (US; to detect the stones, hydronephrosis degree and condition of the parenchyma) and non-enhanced low-dose CT were done. Stone densities and stone size (determined according to the maximum dimension) were measured by CT.

The patients were randomly allocated, by closed envelope, to two groups: Group 1, treated with SWL; and Group 2, treated with mini-URS with laser lithotripsy. The results of both groups were blindly presented to the researcher who did the statistical analysis. Patients with active UTIs were treated first according to culture results and sensitivity.

Preoperative broad-spectrum antibiotic was given to all patients (ceftriaxone 40 mg/kg single intravenous dose).

### Sample size

By assuming that the SFR in SWL was 73.3% [9] and in URS was 98% [10] at 95% CI, for the power of the test to be 80% using Epi info version 6, the number children was calculated to be 62. With 10% added for possible drop out or loss to follow-up, the total number was 68 patients, 34 in each group.

In Group 1, SWL was done in all patients in a supine position with a Dornier lithotripter S (Dornier Medtech, Munich, Germany) under general anaesthesia and fluoroscopic guidance without JJ stenting. Voltage ramping was used with the power between 70% to 90% and a maximum shockwaves number of 3500. The shockwave rate was 70 shocks/min.

In Group 2, URS was performed under general anaesthesia, with the child in lithotomy position. Initially, routine cystoscopy was done in all patients for identification of the target ureteric orifice and insertion of a hydrophilic guidewire under fluoroscopic guidance. We used a 6/7.5 F semi-rigid ureteroscope (Richard Wolf GmbH, Knittlingen, Germany) and isotonic warm saline as the irrigating solution. After approaching the stone, stone disintegration was started using a 200-µm fibre and Ho-YAG laser (sphinx Lisa 100 W, LISA Laser Products GmbH, Katlenburg-Lindau, Germany). The laser setting was set at ‘dusting’ mode (power 7.5–10 W, frequency 20 Hz, energy 0.5 J, and duration 800 ms). A 4.8-F JJ stent (14–20 cm) was placed at the end of the procedure in cases with migrated stones, ureteric perforation, or mucosal injury.

The operative time in Group 1 was the shockwave time, whereas in Group 2 it was calculated starting from the beginning of cystoscopy to the termination of the procedure.

In both the groups, KUB and US was repeated at 2 weeks postoperatively, if a urinoma had formed in cases of ureteric perforation, US-guided drainage was done. The initial SFR after 2 weeks (no stone residual ≥0.3 cm) was calculated. In cases with stone residual of 0.3–1 cm, medical expulsive therapy was used and they were re-evaluated at 1 month postoperatively. Stone residuals of >1 cm or those with complications (obstruction, sepsis, persistent pain or oliguria) were managed accordingly either with SWL, URS or JJ stenting.

Follow-up visits were scheduled at 1, 3 and 6 months postoperatively. At each visit; urine analysis, urine culture and US were done. A contrast study was done if ureteric stricture was suspected due to increased hydronephrosis on US.

Stone-free status (no stone residual ≥0.3 cm) was calculated after 1 month from the initial procedure without the need for auxiliary manoeuvres. Clavien–Dindo Grades were used to categorise complications in our study [11]. The primary endpoint was the SFR and the secondary endpoints were the complications rates in the two groups (Clavien-Dindo Grade ≥III), adjunctive procedures, operative time, and the re-treatment rates.

An adjunctive procedure, was a procedure needed to complete the original procedure or to treat intraoperative complications, where the re-treatment rate comprised the total number of procedures needed to reach a stone-free status after documenting a stone residual with the initial procedure either using the same initial technique or an auxiliary procedure. The efficiency quotient [EQ; where EQ = number of stone-free patients/total number of procedures (including primary treatment, re-treatments and ancillary treatments)] was used to calculate and compare the re-treatment rates between the two groups.

### Statistics

Data analysis was performed using the Statistical Package for the Social Sciences (SPSS®), version 20 (SPSS Inc., IBM Corp., Armonk, NY, USA). Levene’s test was used to measure homogeneity of the two groups. The chi-square test, Fisher’s exact test and Mann–Whitney *U*-test were used as appropriate.

## Results

**Figure 1.**
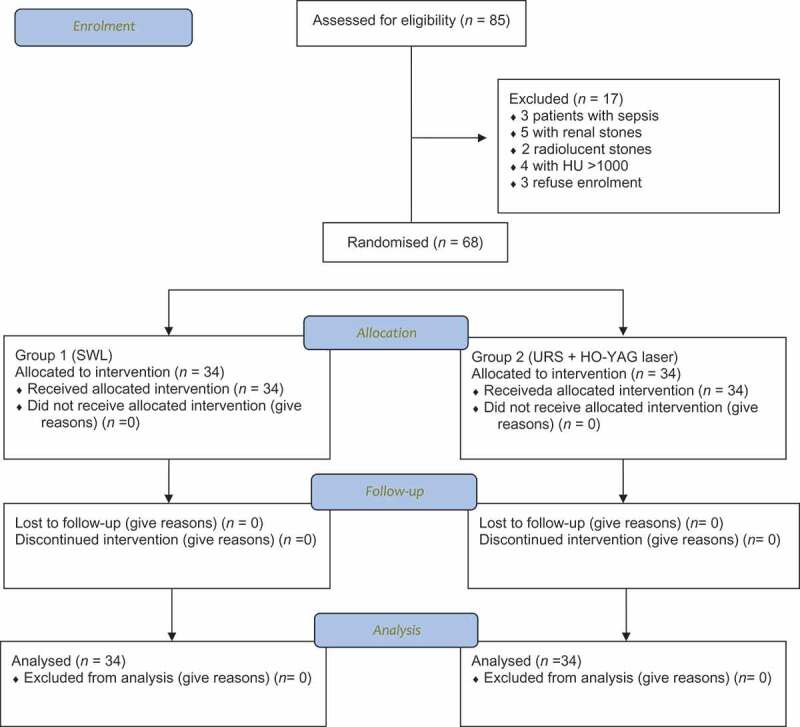
Consolidated Standards of Reporting Trials (CONSORT) flow diagram.

Between January 2015 and March 2019, 85 children presented to our outpatient clinics with UUT ureteric stones of >1 cm. From them, 68 patients met our inclusion criteria (Figure 1). Patients and stones demographics are summarised and compared in Table 1. There were no statistical differences between the two groups for patients or stone characteristics (Levene’s test). The preoperative urine analysis revealed UTIs in 13 cases (seven in Group 1 and six in Group 2) and they were all treated according to culture and sensitivity, and sterile urine was confirmed with a post-treatment culture and sensitivity.

**Table 1.** Patient and stone characteristics.

Variable	Group 1 (SWL)	Group 2 (URS)	Levene’s test	*P*
Age, years, range	3.17–15.25	2.33–15.67	*F *= 1.96	
Mean (SD)	9.4 (4.1)	8.3 (3.6)	*P* = 0.169	
Median	9.10	7.7	Homogeneity	
Mode	5.4	3.6		
Male/female, *n*	20/14	22/12		0.80
Right/left, *n*	18/16	15/19		0.62
Stone size, cm, range	1.2–2.8	1.1–2.6	*F *= 0.002	
Mean (SD)	1.96 (0.43)	1.96 (0.42)	*P *= 0.96	
Median	1.9	2	Homogeneity	
Mode	1.9	2		
Stone density, HU			*F *= 0.371	
Mean (SD)	762 (124.8)	758 (117.5)	*P* = 0.545	
Median	785	753	Homogeneity	
Mode	850	750		
Hydronephrosis, *n*				
Grade 1	12	14		The z-score 0.730
Grade 2	15	16		0.46
Grade 3	7	4	*F* = 0.051	
Median	2	2	*P *= 0.822	
Mode	2	2	Homogeneity

In Group 1, all patients were discharged on the same day, i.e. an outpatient procedure. The mean (SD, range) operative time was 41.9 (3.73, 5–49) min. The number of shockwaves given ranged from 3000 to 3500 in the session. The SFR in Group 1 was 26/34 (76.4%). To reach a stone-free status in all these patients, 12 additional procedures (10 SWL and two URS) were needed. Nine patients (26.4%) were complicated with, haematuria in eight (23.5%), fever without UTI in four (11.8%), steinstrasse in three (8.8%), and UTI + fever in three (8.8%). More than one complication occurred in the same patient. One of the cases with steinstrasse was managed by SWL of the leading stone and the other two needed URS with JJ stenting; one after failed SWL and one for obstruction with UTI and fever (Table 2). Five cases had residual stone in the upper ureter; two of them needed one additional session of SWL and three needed two additional sessions (Table 3).

**Table 2.** Complications and adjunctive procedures of the two groups.

	Group 1 (SWL)		Group 2 (URS)		
Clavien-Dindo Grade	*N*	Description	*N*	Description	*P*
I	8	Haematuria (transient)	5	Haematuria (transient)	
	4	Fever	4	Fever	
II	3	UTI+ fever	1	UTI	
IIIa	0	0	0	0	0.642#
IIIb	3	Steinstrasse	3	2 perforation 1 stone migration	
IV	0	0	0	0	
V	0	0	0	0	
Complicated cases, *n*	9		8		0.780#
Adjunctive procedures, *n*	0		3		0.239*

#Chi-square test; *Fisher’s exact test

**Table 3.** Re-treatment procedures in both groups.

	Group 1 (SWL)	Group 2 (URS)
Number of cases with residual stone	8	1
Description	5 residual stone in upper ureter 3 steinstrasse	migrated stone
Procedures need to achieve 100% SFR		
Number	12	2
Type	10 SWL sessions 2 URS and JJ stent	2 sessions of SWL

In Group 2, the mean (SD, range) operative time was 41.2 (8.3, 27–55) min. The mean (SD, range) laser time was 17.4 (4.2, 9–25) min. No ureteric orifice dilatation was needed, no ureteric access sheath was used, and there was no failure to reach the stones in the upper ureter. The JJ stent was fixed in three patients (adjunctive procedure); one with stone migration and in two cases with perforation. The mean (range) hospital stay was 1.4 (1–3) days (median 1 day). The SFR in Group 2 was 33/34 (97.1%).

Eight patients (23.5%) had 13 complications; fever in four cases (11.8%), haematuria in five cases (14.7%), UTI in one (3.4%), two ureteric perforations (5.8%), and one had stone migration (2.9%). The migrated stone was managed by two sessions of SWL (auxiliary procedure) (Table 3). There were no significant differences between either the number of complicated cases and complications of Clavien–Dindo Grade ≥III (*P* = 0.78 and *P* = 0.64, respectively).

Complications according to the Clavien–Dindo grading system are summarised and compared in Table 2. There were no changes in the SFR between that at 2 weeks and after 1 month.

The mean (SD, range) follow-up was 8.9 (2.2, 6–13) months. There were no cases complicated with stricture in the upper ureter at the 6-month follow-up.

There were significant statistical differences between the two groups for SFR [76.4% (26/34) in Group 1 and 97.1% (33/34) in Group 2, *P* = 0.03] and in the re-treatment rate (*P* = 0.014). The EQ in Group 1 (SWL) was 0.74 and in Group 2 it was 0.94 (URS).

There were no significant statistical differences between operative time between the groups (*P* = 0.653), adjunctive procedures (*P* = 0.239), or the number of complicated cases (*P* = 0.779).

## Discussion

There are many options for the management of UUT ureteric stones in the paediatric age group, e.g. SWL, URS and open surgery.

Paediatric ureters have the capability to pass stone fragments after SWL more efficiently than those of the adult. But, SWL has the prospect of the need of more than one session or the need of additional manoeuvres such as JJ stenting or URS.

URS management of stone disease in children became a standard technique due to advances in miniaturised instruments, newly introduced lithotripsy technologies such as lasers, and the development of age- and size-fashioned equipment [8].

Despite wide acceptance in the literature that flexible URS along with laser lithotripsy is the first-line management for ureteric stone diseases in the paediatric age group; the use of semi-rigid URS for paediatric ureteric stones is still an option, especially when miniature URS and laser lithotripsy are available [12].

Drake et al. [13], in their systematic review of the benefits and harms of URS vs SWL in the treatment of UUT ureteric stones, only 22 studies compared URS and SWL out of the 47 that met their inclusion criteria. Of these 22 studies, only four were randomised controlled trials (RCTs) and one was a quasi RCT, none of them was in a paediatric-age population. They concluded that, URS was superior to SWL in the terms of SFR and re-treatment rates in most of the studies, whereas it was inferior for hospital stay, adjunctive procedures and complications rates. Also, higher complications rates across all Clavien–Dindo grades were reported when URS was used for UUT ureteric stones [13]. These higher complications rates and grades were obvious in most of the reviewed studies except in two, where the authors reported that stone migration and steinstrasse with SWL, which required JJ stenting (Grade III), were more [14,15].

In our present study, the initial SFR (after 2 weeks) and that after 1 month were the same, 76.4% in Group 1 and 97.1% in Group 2 (*P* = 0.012). Drake et al. [13] reported significant differences in favour of URS in nine studies and no significant differences in 13. In these studies the time-point at which SFR was measured varied between immediately and 4-weeks postoperatively. Management of UUT ureteric stones with URS has a higher SFR than SWL regardless of the stone size, as reported by the AUA guidelines in 2016 [16,17]. Our present SFR in the URS group (Group 2) was better than that reported in many studies. Atar et al. [8] reported a success rate of 78.6% using a 7.5-F semi-rigid ureteroscope and 92.6% using a 4.5-F semi-rigid ureteroscope with a significant difference only found in patients aged <3 years [66.7% in Group 1 (7.5 F) and 93.8% in Group 2 (4.5 F), *P* < 0.05].

Yucel et al. [12] using 7.5-F semi-rigid URS with pneumatic lithotripsy reported a success rate of 84.3% in 48 patients, 17 of them had stones in the upper ureter. Three of the patients with UUT ureteric stones (17.6%) needed SWL for migrating stones. Thomas et al. [18] had an 88% SFR in 29 patients with stones located from the renal pelvis down to the lower ureter using URS with laser lithotripsy. In three patients with proximal ureteric stones, two URS and one SWL were needed to achieve a stone-free status. Raza et al. [19] used rigid URS with holmium laser lithotripsy in seven patients, with mean stone size of 1.0 cm, and reported a SFR of 100% with no complications.

Minevich et al. [10] reported a similar SFR to our present study, with a 98% success rate for URS in 58 patients with ureteric and renal stones (16 had UUT ureteric stones, seven renal stones) with one patient (1.3%) developing lower ureteric stricture that needed laser incision. Smaldone et al. [20] reported an increase in the rate of URS for the management of paediatric renal stones of seven-fold during the period 2001–2005. In their study of >100 patients (19% of them had UUT ureteric stones), the SFR was 91% and five patients needed stenting for perforation or extravasation and one was complicated by stricture. Mursi et al. [21] found patients aged ≤2 years, stone size >1.5 cm and a location of the stone in the renal pelvis significantly increased the failure and complication rates, with a success rate of 85% and 53% for UUT ureteric and renal pelvis stones, respectively.

Success rates for SWL for UUT ureteric stones vary widely in reported series between 57% and 96% [22–24]. The AUA in 2007 found a comparable SFR between SWL and URS for UUT ureteric stones >1 cm of 79% vs 74% [25]. In 2016, the AUA recommended either URS or SWL for paediatric patients with UUT ureteric stones not likely to pass and medical expulsive therapy for stones of <1.0 cm [15].

Re-treatment (including the same procedure or an auxiliary one) was needed in 23.5% of cases in Group 1 (SWL) and in 2.9% in Group 2 (URS) (*P* = 0.014). Auxiliary SWL was needed in one case (two sessions) in Group 2 due to migration of stones in the lower calyx; while in Group 1, 10 sessions of SWL were needed in seven cases; five cases with residual stones in the upper ureter and two steinstrasse cases, and URS was needed in two cases with steinstrasse (one of them after failed SWL). Drake et al. [13] reported re-treatment rates ranged from 0% to 61.1% for SWL and from 0% to 18% for URS, with significance differences in two of the three studies using *P* values in the evaluation of URS vs SWL. The total number of procedures needed to reach a 100% stone-free status (EQ) was significantly lower in the URS group (*P* = 0.014). The re-treatment rate in our present URS group was lower than other studies, which ranged from 5% to 22% [4,8,12,18]. In Group 1, 18 complications occurred in nine patients and in Group 2 eight patients had 13 complications (*P* = 0.78). Drake et al. [13] found significantly higher complication rates with URS in eight studies, with SWL in two studies, and a non-significant difference in five studies. They also noted that Grade ≥III complications were more prevalent with URS than with SWL. Ureteric injury was not reported with SWL and varied with URS between 0% and 6.6%. In both the present groups, complications ranged from Grade I to IIIb on Clavien–Dindo classification, in Group 1, steinstrasse was the most morbid complication and occurred in three cases (8.8%) but the commonest was haematuria in eight patients (23.5%).

In Group 2, two patients had perforations that necessitated JJ insertion. There was no significant difference between Grade ≥III complications in the two groups (*P* = 0.64). Yucel et al. [10] reported a complication rate of 14.8% of 48 patients, 17 of them with mid and proximal ureteric stones. Severity of complications varied among similar studies, ranging from no complications or just haematuria in some series [17,19] to ureteric perforation necessitating JJ stent insertion in others [8,18].

Our present mean (SD) operative time in Group 2 was 41.2 (8.3) min; which was shorter than that reported by Atar et al. [8] at 53 min. Yucel et al. [12] had a longer operative time, with a mean of 65 min (use pneumatic lithotripsy). In Group 1, the mean (SD) operative time was 41.9 (3.7) min with no significant difference between the two groups (*P* = 0.65).

The limitations of our present study include the small sample size, exclusion of high stone density and radiolucent stones, and the determination of stone size according to the maximum diameter instead of stone burden or volumetric assessment. Another limitation of the present study was the evaluation of the stone residuals by US and KUB, which can miss some small stone residual.

## Conclusion

Miniature semi-rigid URS with Ho-YAG laser lithotripsy was more effective in UUT ureteric stones >1 cm than SWL, with no increased risk of complications either in their number or the grade.
